# Effect of rate of pyrolysis on the textural properties of naturally-templated porous carbons from alginic acid

**DOI:** 10.1016/j.jaap.2016.07.002

**Published:** 2016-09

**Authors:** Andrew S. Marriott, Andrew J. Hunt, Ed Bergström, Jane Thomas-Oates, James H. Clark

**Affiliations:** a Bristol-Myers Squibb, Reeds Lane, Moreton, CH46 1QW, UK; b Green Chemistry Centre of Excellence, Department of Chemistry, The University of York, Heslington, York, North Yorkshire, UK; c Centre of Excellence in Mass Spectrometry and Department of Chemistry, The University of York, Heslington, York, North Yorkshire, UK

**Keywords:** Biomass-derived porous carbons, Pyrolysis rate, N_2_ porosimetry, Thermal gravimetry, Mesoporosity

## Abstract

The effect of pyrolysis rate on the properties of alginic acid-derived carbonaceous materials, termed Starbon^®^, was investigated. Thermal Gravimetry-IR was used to prepare porous carbons up to 800 °C at several rates and highlighted increased CO_2_ production at higher pyrolysis rates. N_2_ porosimetry of the resultant carbons shows how pyrolysis rate affects both the mesopore structure and thus surface area and surface energy. Surface capacity of these carbons was analysed by methylene blue dye adsorption. In general, as the rate of pyrolysis increased, the mesopore content and adsorbent capacity decreased. It is considered here that the rapid production of volatiles at these higher rates causes structural collapse of the non-templated pore network. The work here demonstrates that pyrolysis rate is a key variable which needs to be controlled to maximise the textural properties of Starbon^®^ required for adsorption applications.

## Highlights

• Textural properties studied by N_2_ porosimetry and thermal gravimetric analysis.
• Mesoporosity of Starbon^®^ decreases with increasing rate of pyrolysis.
• Porosity collapse a result of rapid volatile compound generation during fast pyrolysis.
• Increased pyrolysis rate decreases adsorption capacity of Starbon^®^.

## Graphical abstract

**Figure:**
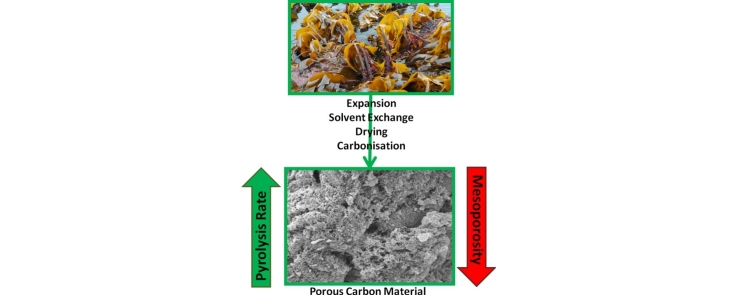

## 1 Introduction

Adsorption is the most common treatment employed by industry to clean process waste streams, with activated carbon the most widely used sorbent material in wastewater treatment [1]. Questions arise about the cost-effectiveness of using activated carbons, due to problems with regenerating the activated surface after use, combined with the high costs of manufacturing the materials. There is also a growing need to recover adsorbed species, consistent with the circular economy, and this is very difficult with microporous carbons. Consequently, a lot of attention geared towards finding low-cost materials for the synthesis of activated mesoporous carbons has been invested. Sources like the by-products of timber and agricultural industries, food waste (*e.g.* pecan shells [2] and rice husks [3]) as well as activated sludges have been studied in the search for effective and affordable adsorbent materials [4].

Alginic acid is a marine polysaccharide mainly produced by brown algae. It consists of β-1,4-linked d-mannuronic acid and α-1,4-linked l-guluronic acid residues arranged in homopolymeric blocks separated by regions of alternating sequences of the two monomers. When added to water, alginic acid forms a mechanically stable porous hydrogel which after subsequent solvent exchange with an organic solvent can be dried using supercritical CO_2_ to form an aerogel with high surface area and mesoporosity [5]. Recent work has shown how pyrolysis of the aerogel leads to the formation of a mesoporous carbon material (termed Starbon^®^) with which adsorption and separations applications have been examined [6], [7], [8]. In particular Parker et al. used Starbon^®^ for the adsorption of dyes, reporting that dye capacity and speed of adsorption were found to be superior to those of the commercial activated carbon Norit using Starbon^®^ pyrolysed to 800 °C [8]. Maintaining the mesoporous structure of Starbon^®^ is key to its performance as an adsorbent and separation medium and understanding the effect of pyrolysis on the aerogel structure is paramount in the development of a consistent porous carbon. This paper describes the effect of pyrolysis heating rate on the pore structure of alginic acid-derived Starbon^®^ as well as the adsorption capabilities of the resultant materials.

## 2 Experimental

### 2.1 Synthesis of alginic acid-derived aerogel materials

Alginic acid from brown algae (Sigma Aldrich, UK) was gelatinised in distilled water (1 g:20 mL alginic acid:water ratio) and stirred for 2 h at 90 °C. The resulting sol gel was then retrograded at 5 °C for 24 h. The water was exchanged for ethanol and the resulting alcogel dried in supercritical CO_2_ (scCO_2_). Drying was conducted using a Thar SFE-500 supercritical extractor heated to 40 °C and held at a pressure of 120 Bar for 2 h under dynamic flow conditions (40 g min**^−^**^1^ CO_2_). The extractor was subsequently allowed to depressurise over a period of 12 h to give the final alginate aerogel.

### 2.2 Thermal gravimetry and preparation of alginic acid-derived porous carbon (Starbon^®^) materials

Thermal gravimetry (TG) and TG-Infrared (TG-IR) spectroscopy was performed using a Netzsch 409 thermal gravimetric system coupled *via* a heated transfer pipe to a Bruker Equinox 55 FT-IR spectrometer. Data was collected in chromatography mode set at 64 scans per spectrum. The spectrometer resolution was 4 cm**^−^**^1^. The final carbon samples were prepared using 500 mg dried aerogel per analysis and all samples pyrolysed to 800 **°**C under an inert nitrogen atmosphere in the thermogravimetric system. Samples were prepared at ramped heating rates of 1–3, 5–7, 10 and 20 K min**^−^**^1^. This was followed by a 10 min final temperature hold and controlled sample cooling over 2 h by flow of nitrogen gas. Yields of ca. 20% for the resulting alginic acid-derived Starbon^®^ material were consistently generated across all the heating rates tested (data not shown).

### 2.3 N_2_ adsorption-desorption experiments

N_2_ adsorption-desorption porosimetry of the pyrolysed samples (80 mg) was performed at −196 °C using a Micrometrics ASAP 2010 porosimeter with samples dried under vacuum for 4 h at 90 °C to remove any residual moisture prior to analysis. Figures were generated using OriginPro 8 software.

### 2.4 Methylene blue adsorption study

Activated charcoal (Norit—Fluka, UK) was washed with deionised water at 80 °C, filtered and vacuum oven-dried prior to use. A 10 mg L**^−^**^1^ standard stock solution of methylene blue dye (Alfa Aesar, UK) was prepared in a 2 L volumetric flask using deionised water. Sample material (5 mg) was weighed into a sample tube which was then filled with 25 mL of the stock dye solution and stirred for 24 h at room temperature. Due to complete adsorption of dye by the 1 and 2 K min**^−^**^1^ samples at this scale an additional experiment was performed with these materials where 5 mg of sample material was stirred in 100 mL stock standard solution in a 100 mL volumetric flask. The non-adsorbed dye solution was filtered using 0.45 μm PTFE filter and UV–vis analysis performed using a Jasco V-550 UV–vis spectrometer at 664 nm. Linearity solutions of methylene blue at 1, 2.5, 5, 7.5 and 10 mg L**^−^**^1^ were used to generate a 5-point adsorption calibration curve (R^2^ = 0.998).

## 3 Results and discussion

### 3.1 N_2_ porosimetry analysis

**Fig. 1:**
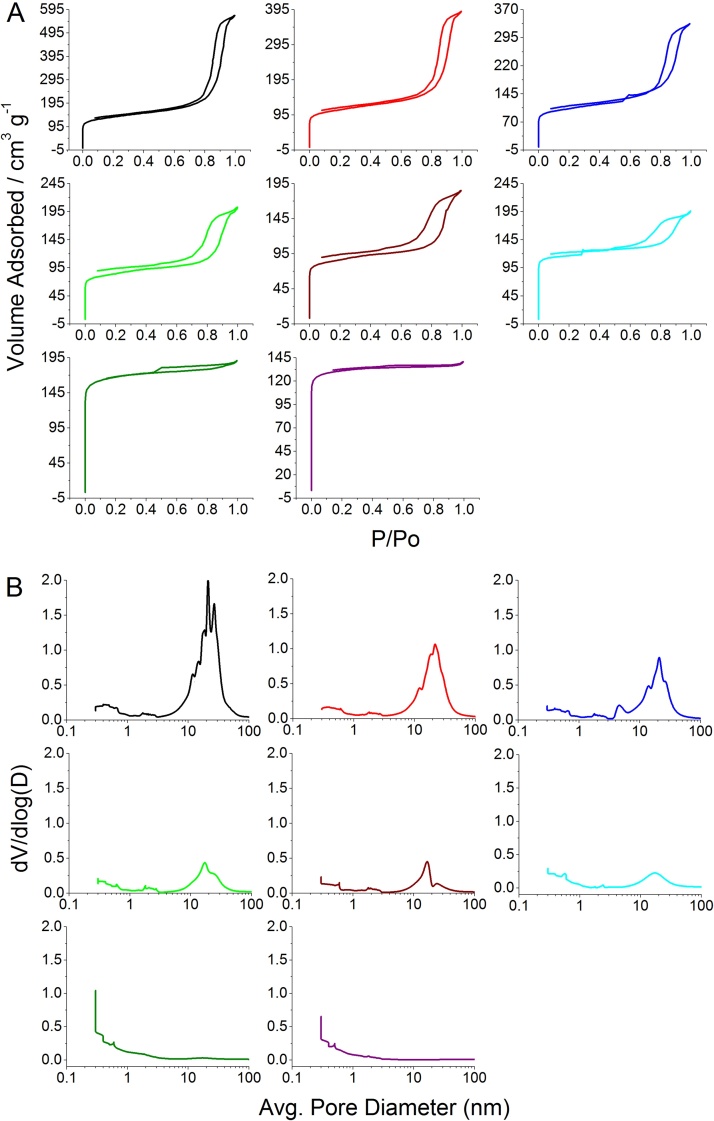
(A) N_2_ sorption isotherm plots and (B) corresponding adsorption pore size distribution. The plots are arranged in ascending order of pyrolysis rate, represented by the following colours: 1 K min^−1^ = Black; 2 K min^−1^ = Red; 3 K min^−1^ = Blue; 5 K min^−1^ = Light Green; 6 K min^−1^ = Brown; 7 K min^−1^ = Cyan; 10 K min^−1^ = Dark Green; 20 K min^−1^ = Purple. (For interpretation of the references to colour in this figure legend, the reader is referred to the web version of this article).

Table 1 summarises the N_2_ sorption data collected for each sample. The main observations are the decrease in both desorption pore volume and average pore diameter, suggesting that there is a decline in mesoporosity as the rate of pyrolysis is increased. This trend is visualised by comparing pore size distribution plots (Fig. 1(A)); here, dV/dlog(D) at a given pore diameter is directly related to the volume of nitrogen gas adsorbed. For materials formed at low rates of pyrolysis a significant proportion of nitrogen is adsorbed in the mesopore/low macropore region (2–50 nm) in the form of a peak centred around 20–30 nm. As pyrolysis rates increase, this peak volume reduces and shifts towards the micropore region such that when the precursor is pyrolysed at 10 K min^−1^ the pore size distribution of the resulting material is shifted towards the micropore region.

N_2_ sorption isotherm plots for Starbon^®^ samples generated at each of the different heating rates (Fig. 1(B)) provide further evidence of this general trend. The isotherm plots show a progression of the volume of N_2_ adsorbed at the material surface as the system partial pressure (P/P_o_) is increased from 0 to 1 and then subsequently desorbed leading to the Type IV hysteresis loops observed. Fig. 1(B) shows how the amount of N_2_ adsorbed by meso/macropores, represented by the rise in the volume of gas adsorbed above ca. 0.8 P/P_o_, evidently reduces as the heating rate is increased. It is also noted that the hysteresis loops broaden as mesoporosity reduces indicating that the external pore openings are narrower in materials generated at higher pyrolysis rates.

The characteristic energy of a material determined by the Dubinin-Radushkevich equation, correlates to the adsorption potential of that material and these values for the pyrolysed Starbon^®^ materials are also given in Table 1 [9]. Here, characteristic energy values (CE_D-R_) tend to increase with increasing rates of heating with a marked change in these values between 6 and 7 K min**^−^**^1^.

This adsorption potential step change, between the materials formed at 6 and 7 K min**^−^**^1^ may be due to the large increase in microporosity between these samples. The narrow gap between the walls of a micropore can lead to the overlap of adsorption potential thus intensifying the attractive forces at these pores and enhancing adsorption potential above that theoretically expected [10]. The BET surface area (S_BET_) difference between the sample pyrolysed at 6 K min**^−^**^1^ and those pyrolysed at 7 K min**^−^**^1^ and above is considerable, whilst supporting data from the pore size distribution plots for these higher heating rate samples highlight the loss of mesoporosity and an almost complete shift to a microporous carbon product.

The rise in micropore content is likely due to the collapse of micro/mesopore structure during pyrolysis and could result in the overall adsorption potential step change between the sample pyrolysed at 6 K min**^−^**^1^ and those pyrolysed at higher rates. Although this would suggest that Starbon^®^ pyrolysed at higher pyrolysis rates would yield much stronger adsorbents, the narrow pore diameter of micropores would limit the range of adsorbate classes which could be effectively adsorbed, to small gaseous molecules. Although beyond the scope of this work, further study using density functional theory methodology would allow for a more detailed characterization of the variations in pore size distribution and pore morphology at the micropore/mesopore level as a consequence to increasing the rate of pyrolysis [11], [12].

### 3.2 Thermal gravimetric analysis

**Fig. 2:**
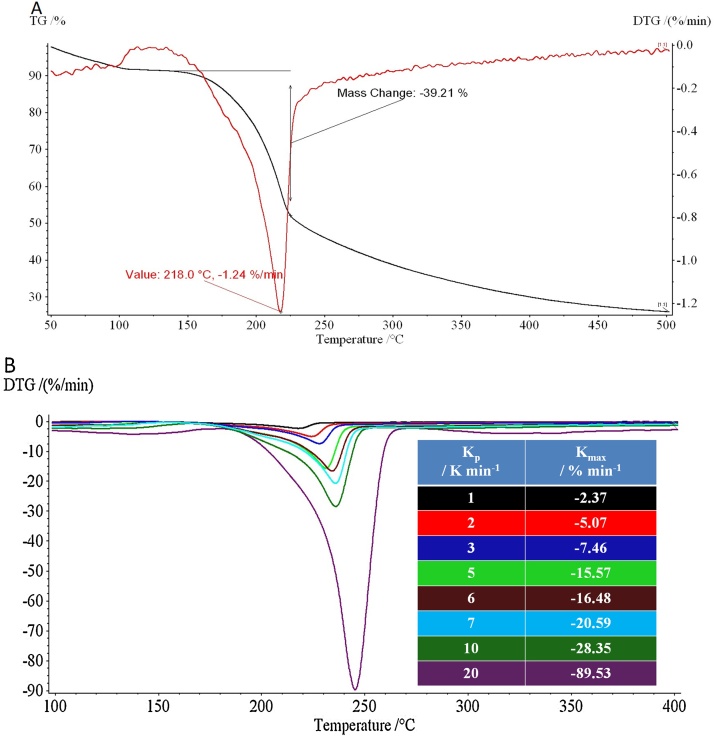
(A) Representative thermal gravimetry (TG-black line) and differential TG (DTG-red line) plot (B) DTG plots for all samples with table showing K_max_, the maximum rate of mass loss per sample. (For interpretation of the references to colour in this figure legend, the reader is referred to the web version of this article).

In order to study the pyrolysis process and how the rate of heating affects the material during the pyrolytic formation of Starbon^®^, data from TG, differential TG (DTG) and TG coupled to a Fourier-transform infrared spectrometer (TG-IR), recorded during the pyrolysis process, were examined. Fig. 2(A) provides a representative profile of the TG and DTG data collected during pyrolysis at 1 K min^−1^, the remaining heating rates showed very similar profiles. The major feature of the TG trace at around 220 °C is a significant decomposition step which results in mass loss of 30–40%. This step is associated with the decarboxylation of the uronic acid residues [13], [14]. Above 220 °C further gradual mass loss was observed, associated with the conversion of intermediate olefinic groups to an aromatic (fullerene-like) carbon by 800 °C which has been studied elsewhere [6], [15].

**Fig. 3:**
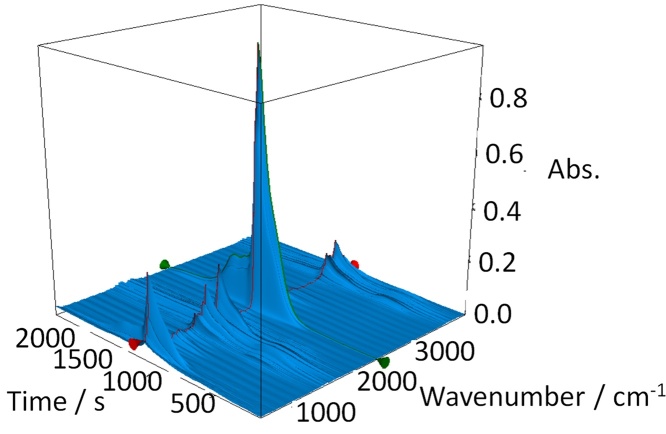
Thermal gravimetry infra-red spectroscopy (TG-IR) time-series of alginic acid-derived Starbon^®^ prepared at 10 K min^−1^.

Fig. 2(B) plots the DTG data for all samples, where the maximum rate of decarboxylation (K_max_) is determined at the apex of the DTG peak. Increasing the rate of pyrolysis results in a corresponding rise in K_max_, which in turn implies that there is a greater volume of volatile compounds generated per minute. This was confirmed by TG-IR analysis which was used to record the volatile components emitted from the sample. Fig. 3 shows the TG-IR time-series for the Starbon^®^ sample pyrolysed at 10 K min**^−^**^1^ which is dominated by the intense asymmetric stretch band of carbon dioxide (ca. 2300 cm**^−^**^1^) released on decarboxylation. TG-IR at slower rates of pyrolysis (2 K min**^−^**^1^ and 5 K min**^−^**^1^) produced similar plots, although the intensity of the carbon dioxide stretch was much reduced (see Supplemental Data Figs. S1 and S2 respectively) indicating that the rate of CO_2_ evolution was much lower in these samples.

The generation of increasing volumes of volatile molecules as the rate of pyrolysis increases suggests that greater pressure would be exerted on the pore network of these materials and that they are increasingly prone to structural collapse. Coupled to this, it has previously been reported that the pyrolysis of expanded polysaccharides in the presence of water causes the collapse of the pore networks [16], [17]. This is due to the high surface tension of water generated at the hydrophilic polysaccharide pore walls which, when volatilised, exerts sufficient force to weaken and thus collapse the pore structure. Although the water in our preparation was exchanged for an organic solvent and then dried (scCO_2_), water may still be present due to residual surface tension in the narrow, polar micropore networks which are never fully removed from non-templated porous carbons [5]. The loss of this tightly bound water vapour, combined with additional water loss generated from the breakdown of the carbohydrate residues’ hydroxyl groups was observed with increased intensity of the 3600–3200 cm**^−^**^1^ region at the time of CO_2_ release. At fast pyrolysis rates it is conceivable that sufficient water vapour is generated to weaken the sample pore structure. This, combined with the pressure arising from the rapid production of volatiles are believed to be the factors which effect the Starbon^®^ pore structure during fast pyrolysis.

### 3.3 Methylene blue adsorption study

Hsieh and Teng reported how increasing the proportion of the total pore volume that was due to mesopores enhanced the adsorption capacities of activated carbons, determined by comparing carbons with differing mesopore content but similar total pore volumes [9]. Therefore, it is proposed that the adsorption capacity of the Starbon^®^ materials is likewise dependent on the percentage of mesopores.

An adsorption study using the dye molecule methylene blue was performed in order to determine how material adsorption capacity may be altered by the mesopore content of the porous carbon material. The material (5 mg) was stirred in a vessel containing a 10 mg L**^−^**^1^ stock solution of methylene blue for 24 h and the absorbance measured by UV–vis spectroscopy from which the adsorbent capacity was determined.

The data, summarised in Table 2, shows a clear trend of decreasing adsorbent capacity with decreasing sample mesoporosity. The adsorbent coverage value shows the percentage area of the adsorbent that has adsorbed the dye, assuming monolayer coverage. The coverage is larger for materials produced at lower heating rates and may result from the extended mesopore network in these materials. The trend in adsorbent capacity is reversed slightly between samples pyrolysed at 6 K min**^−^**^1^ and 7 K min**^−^**^1^ although this could be due to the large increase in total surface area countering the reductions in both pore diameter and pore volume. The adsorbent capacity calculated for Norit (39 mg g**^−^**^1^) was within 10% of that reported by Parker et al. (42 mg g**^−^**^1^) [8], analogous to Starbon^®^ pyrolysed at 5 K min**^−^**^1^ but significantly lower than that of the best adsorbing Starbon^®^ (1 K min**^−^**^1^:138 mg g**^−^**^1^).

## 4 Conclusions

In conclusion, we have shown that the rate of pyrolysis has a marked effect on the pore structure of alginic acid-derived Starbon^®^. The internal pressures associated with rapid formation of volatiles at higher pyrolysis rates, as well as the release of water vapour are proposed to be combinatorial effects which reduce mesopore content in these materials. These results highlight how crucial the control of pyrolysis is in order to produce the best materials for adsorption or separation applications.

## Figures and Tables

**Table 1:** N_2_ sorption data for all pyrolysed Starbon^®^ samples.

ak_p_/K min^−1^	bS_BET_/m^2^ g^−1^	cPV_des_/cm^3^ g^−1^	dPD/nm	eCE_D-R_/kJ mol^−1^	fMic./%
1	492	0.78	12.89	23.03	23.2
2	400	0.51	11.42	23.94	27.8
3	385	0.42	10.35	24.77	31.8
5	310	0.21	9.05	23.97	44.2
6	313	0.18	8.18	25.90	48.5
7	434	0.15	7.74	30.22	60.9
10	585	0.07	3.85	31.73	86.8
20	465	0.02	3.64	32.75	93.5

a k_p_ = Pyrolysis heating rate.

b S_BET_ = Brunauer-Emmett-Teller surface area.

c PV_des_ = Barrett-Joyner-Halenda (BJH) desorption pore volume.

d PD = BJH desorption average pore diameter.

e CE_D-R_ = Dubinin-Radushkevich characteristic energy.

f Mic. = Percentage microporosity.

**Table 2:** Adsorbent capacities and surface area dye coverage of methylene blue for all pyrolysed Starbon^®^ samples.

ak_p_/K min^−1^	bAdsorbent Capacity/mg g^−1^	cSurface Area/m^2^ g^−1^	dAdsorbent Coverage/%
1*	>50 (138)	>91 (252)	>18 (51)
2*	>50 (110)	>91 (201)	>22 (50)
3	47	86	22
5	39	71	23
6	25	46	15
7	30	55	13
10	10	18	3
20	9	16	4

a k_p_ = Pyrolysis heating rate.

b Calculated based on the UV–vis adsorbance of methylene blue at 664 nm.

c Based on methylene blue surface area = 1.026 nm^2^.

d Assumes monolayer coverage only.

* Bracketed data based on adsorption in 100 mL stock solution.
