# Supplementary material for: Effect of rate of pyrolysis on the textural properties of naturally-templated porous carbons from alginic acid
Source: J Anal Appl Pyrolysis. 2016 Sep;121:62–6. doi: 10.1016/j.jaap.2016.07.002 (PMC5042340; doi:10.1016/j.jaap.2016.07.002)
Supplement: Supplementary file 1 [file mmc1.pdf]

Supplementary Data

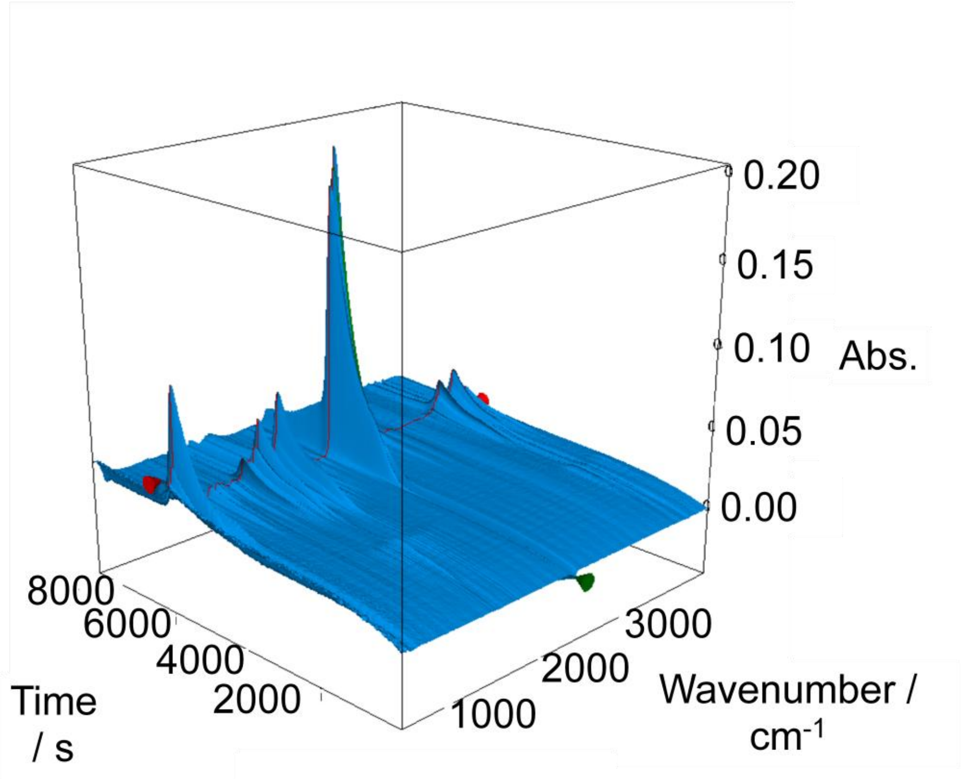

Figure S1: TG-IR time-series of Starbon® prepared at 2 K min^{-1}

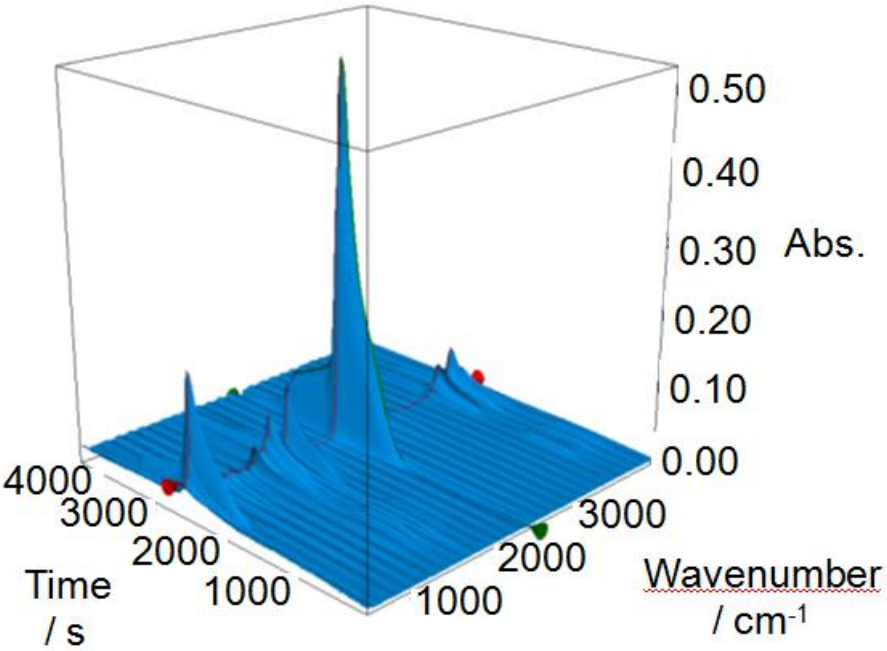

Figure S2: TG-IR time-series of Starbon® prepared at 5 K min^{-1}
